# Supplementary material for: In Vivo Evaluation of Two Hemorrhagic Shock Resuscitation Controllers with Non-Invasive, Intermittent Sensors
Source: Bioengineering (Basel). 2024 Dec 20;11(12):1296. doi: 10.3390/bioengineering11121296 (PMC11727294; doi:10.3390/bioengineering11121296)
Supplement: Supplementary file 1 [file bioengineering-11-01296-s001.zip › bioengineering-3361300-supplementary.pdf]

bioengineering

MDPI

Article

In Vivo Evaluation of Two Hemorrhagic Shock Resuscitation Controllers with Non-Invasive, Intermittent Sensors

Tina M. Rodgers, David Berard, Jose M. Gonzalez, Saul J. Vega, Rachel Gathright, Carlos Bedolla, Evan Ross and Eric J. Snider *

Organ Support and Automation Technologies Group, U.S. Army Institute of Surgical Research, JBSA Fort Sam Houston, San Antonio, TX 78234, USA
* Correspondence: eric.j.snider3.civ@health.mil; Tel.: +1-210-539-8721

Supplementary Materials

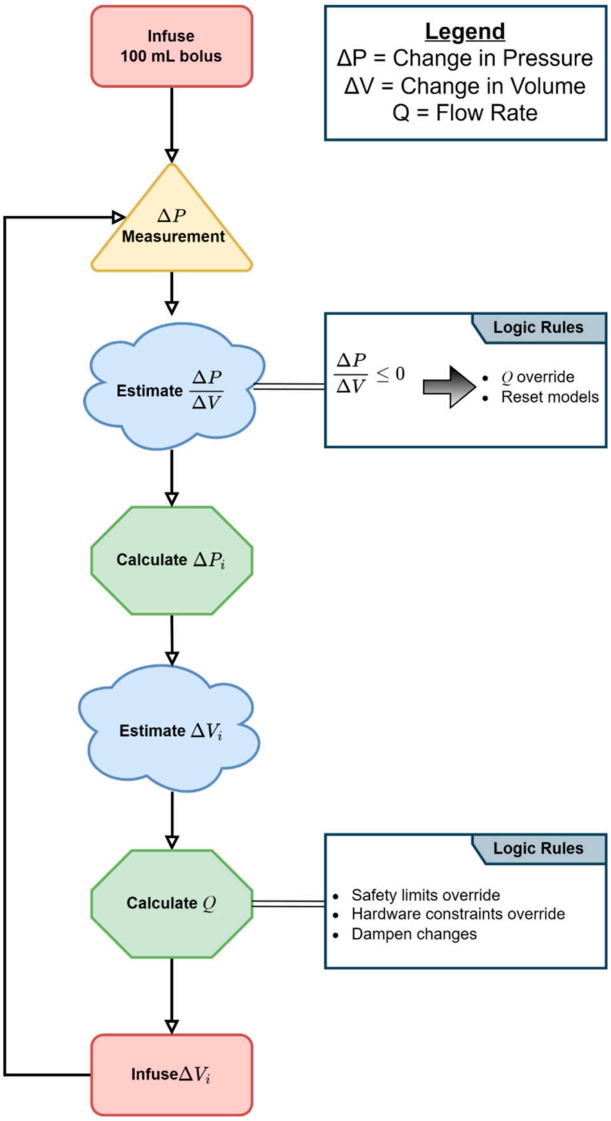

Figure S1. Overview of Adaptive Resuscitation Controller (ARC) Logic. Reproduced from [21].

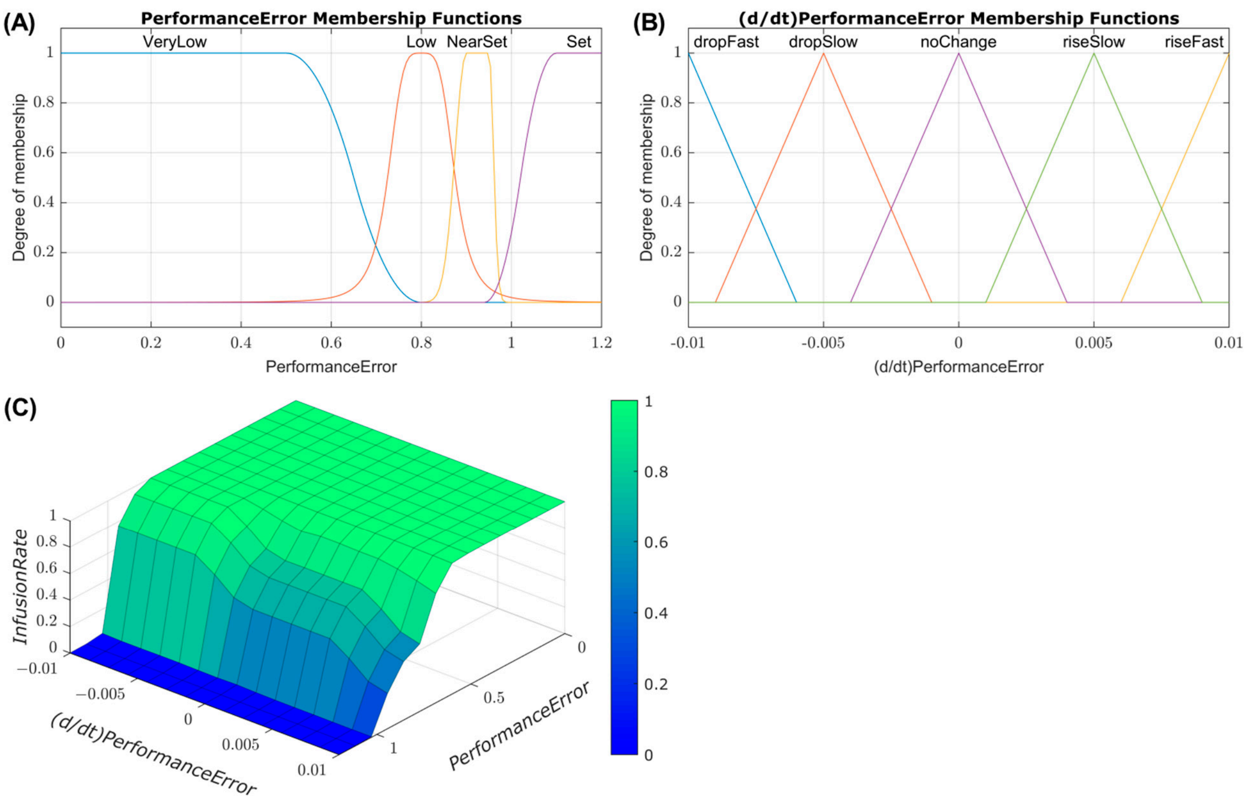

Figure S2. Overview of Dual-Fuzzy Logic (DFL) Controller Design. (A,B) Membership functions for both inputs for the DFL Controller. (C) A surface plot of both DFL input to the controller's output.

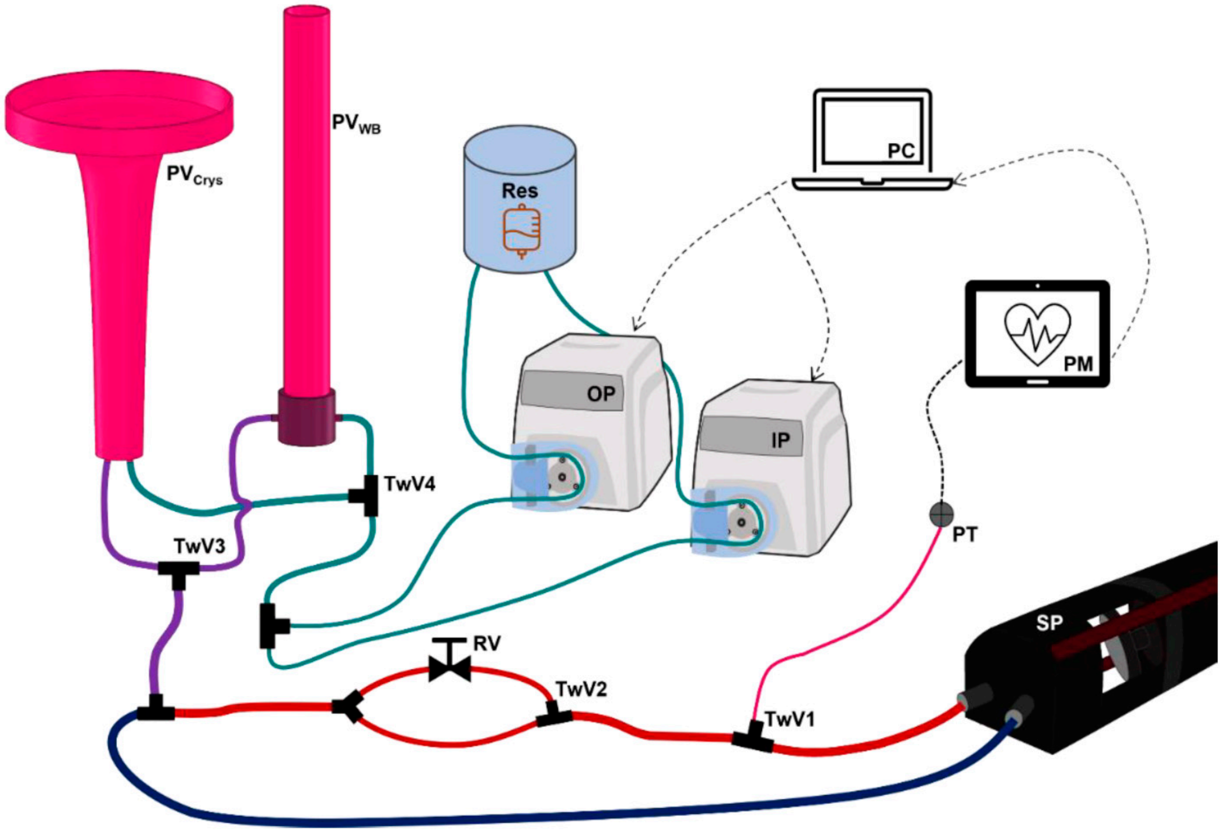

Figure S3. Diagram of the Hardware in Loop Testbed for Evaluation of Resuscitation Controllers. Reproduced from [29].

Table S1. Adaptive Resuscitation Controller Performance Metrics Across Five Swine Subjects.

	Animal 1	Animal 2	Animal 3	Animal 4	Animal 5	MEAN	STD
Median Performance Error (%)	0.00	3.08	0.00	4.62	3.64	2.27	1.91
Median Absolute Performance Error (%)	1.67	3.08	1.54	6.15	3.64	3.21	1.68
Target Overshoot (%)	10.00	20.00	7.69	32.31	20.00	18.00	8.75
Effectiveness (%)	96.67	86.81	94.58	64.58	73.75	83.28	12.33
Resuscitation Effectiveness (%)	96.81	92.92	94.58	93.06	96.81	94.83	1.71
Wobble (%)	1.67	3.08	1.54	3.08	1.82	2.24	0.69
Divergence (%/hr)	4.00	16.71	16.13	4.48	33.48	14.96	10.75
Rise-Time Efficiency (min)	1.42	2.83	2.50	2.67	1.58	2.20	0.58
Median Infusion Rate (mL/min)	1.40	0.00	42.10	0.00	0.00	8.70	16.71
Mean Infusion Rate (mL/min)	41.15	28.29	62.29	8.80	21.42	32.39	18.24
Area Above Target (mmHg x min)	55.00	118.75	48.42	236.17	162.67	124.20	70.11
Area Below Target (mmHg x min)	-59.42	-92.58	-74.17	-82.33	-41.00	-69.90	18.07
Infusion Rate Variability (mL/min)	35.01	29.06	36.15	8.96	19.23	25.68	10.29

Table S2. Dual-Input Fuzzy Logic Controller Performance Metrics Across Five Swine Subjects.

	Animal 1	Animal 2	Animal 3	Animal 4	Animal 5	MEAN	STD
Median Performance Error (%)	3.33	0.00	0.00	-3.08	-1.54	-0.26	2.13
Median Absolute Performance Error (%)	3.33	1.67	1.67	3.08	1.54	2.26	0.78
Target Overshoot (%)	26.67	15.00	23.33	7.69	3.08	15.15	8.96
Effectiveness (%)	80.42	94.58	92.36	92.64	95.83	91.17	5.52
Resuscitation Effectiveness (%)	98.89	96.53	96.11	92.64	95.83	96.00	2.00
Wobble (%)	1.67	1.67	1.67	1.54	1.54	1.62	0.06
Divergence (%/hr)	58.53	7.96	-12.96	13.47	3.21	14.04	23.93
Rise-Time Efficiency (min)	0.67	1.58	1.17	1.83	1.58	1.37	0.41
Median Infusion Rate (mL/min)	0.00	0.00	10.48	21.25	18.99	10.14	9.03
Mean Infusion Rate (mL/min)	18.27	28.00	35.30	49.41	52.74	36.74	12.93
Area Above Target (mmHg x min)	176.08	51.92	55.00	26.50	2.83	62.47	59.88
Area Below Target (mmHg x min)	-20.33	-60.33	-65.25	-153.08	-126.92	-85.18	48.11
Infusion Rate Variability (mL/min)	19.44	25.33	40.50	32.79	33.17	30.25	7.23

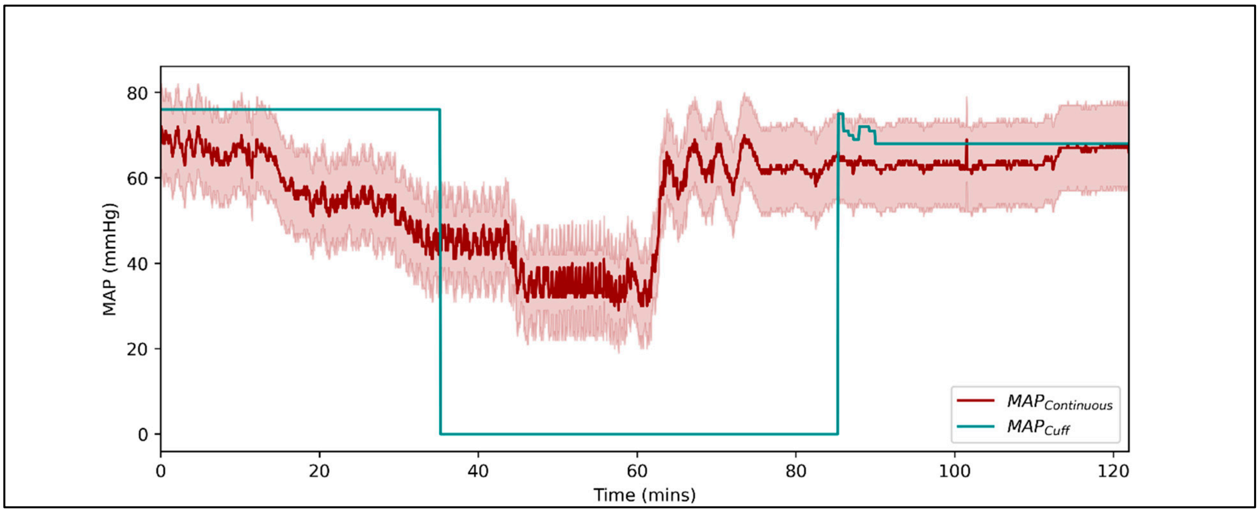

Figure S4. MAP_{Cuff} vs Continuous MAP for a subject result. The shaded region represents the acceptable accuracy range for MAP compared to MAP_{Cuff} and continuous MAP in each example.

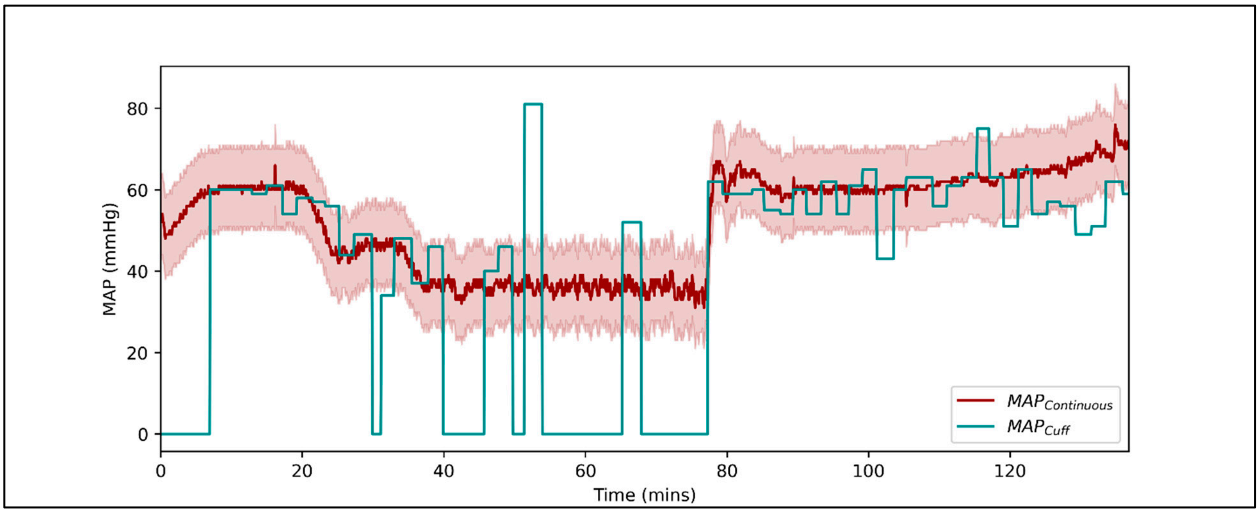

Figure S5. MAPCuff vs Continuous MAP for a subject result. The shaded region represents the acceptable accuracy range for MAP compared to MAPCuff and continuous MAP in each example.

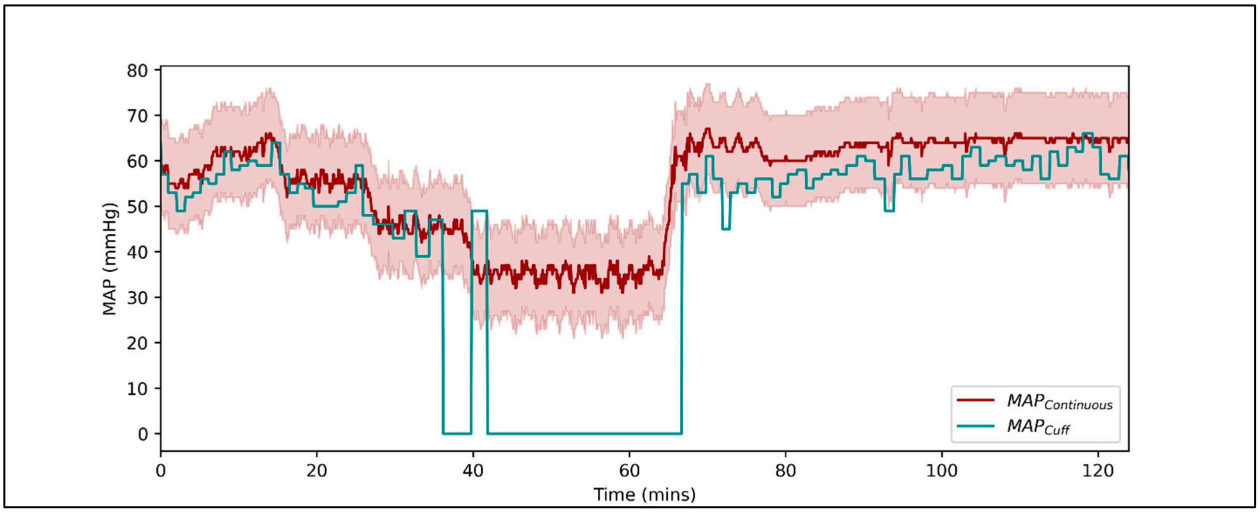

Figure S6. MAPCuff vs Continuous MAP for a subject result. The shaded region represents the acceptable accuracy range for MAP compared to MAPCuff and continuous MAP in each example.

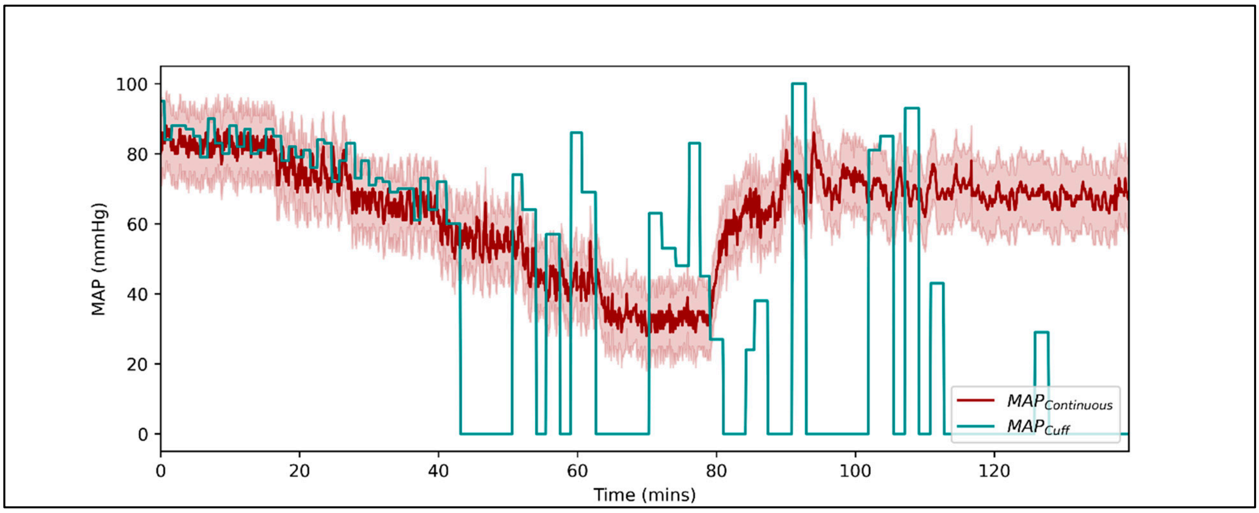

Figure S7. MAPCuff vs Continuous MAP for a subject result. The shaded region represents the acceptable accuracy range for MAP compared to MAPCuff and continuous MAP in each example.

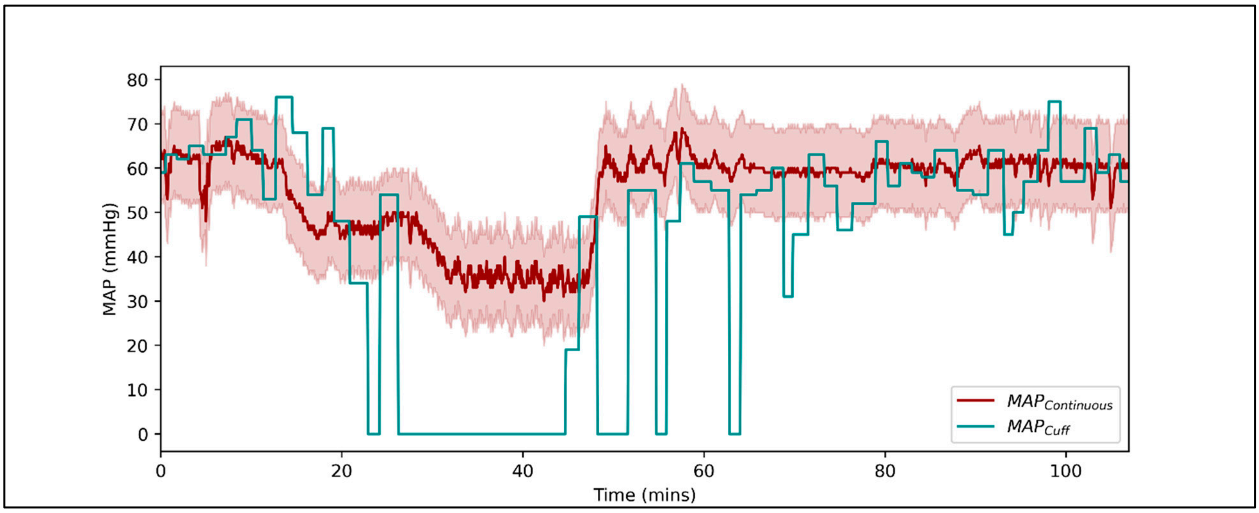

Figure S8. MAP_{Cuff} vs Continuous MAP for a subject result. The shaded region represents the acceptable accuracy range for MAP compared to MAP_{Cuff} and continuous MAP in each example.

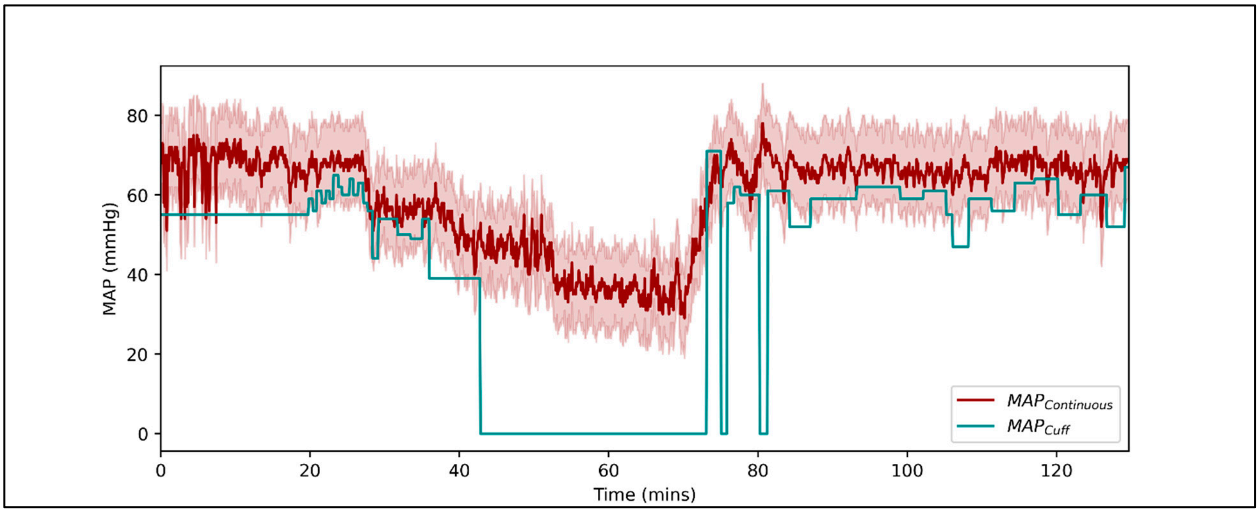

Figure S9. MAP_{Cuff} vs Continuous MAP for a subject result. The shaded region represents the acceptable accuracy range for MAP compared to MAP_{Cuff} and continuous MAP in each example.

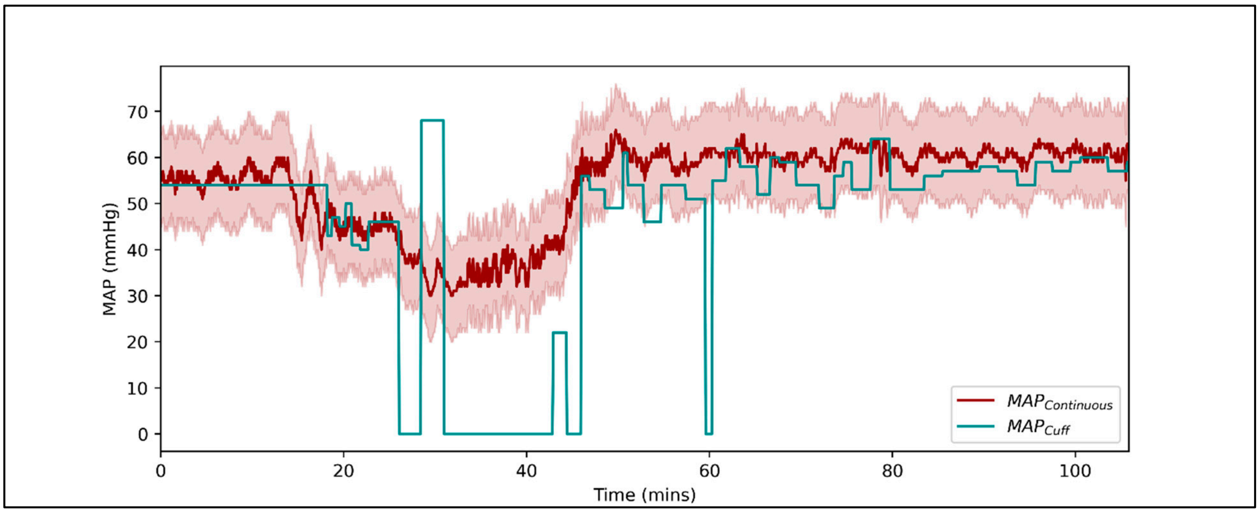

Figure S10. MAP_{Cuff} vs Continuous MAP for a subject result. The shaded region represents the acceptable accuracy range for MAP compared to MAP_{Cuff} and continuous MAP in each example.
